# The impact of sleep disturbances on treatment efficacy and prognosis in patients with depressive and anxiety disorders

**DOI:** 10.3389/fpsyt.2024.1432538

**Published:** 2024-10-14

**Authors:** Qingyu Zhang, Maoqing Tong, Yunxin Ji, Yanbin Hou, Zongze Lou, Danjuan Wu, Yuwei Mi, Pingping Miu, Jiaxin Tian, Zhenzhen Zhu, Liemin Ruan

**Affiliations:** ^1^ Department of Psychosomatic Medicine, the First Affiliated Hospital of Ningbo University, Ningbo, Zhejiang, China; ^2^ School of Medicine, Ningbo University, Ningbo, Zhejiang, China; ^3^ Department of Psychiatry, Affiliated Kangning Hospital of Ningbo University, Ningbo, Zhejiang, China; ^4^ Department of Psychiatry, Ningbo Kangning Hospital, Ningbo, Zhejiang, China

**Keywords:** sleep disturbances, depressive and anxiety disorders, polysomnography, antidepressant therapy, treatment efficacy

## Abstract

**Introduction:**

Little was known about the relationship between sleep disturbances and depressive and anxiety disorders, as well as the efficacy of treatment regimens.

**Methods:**

During 2021 to 2023, a total of 417 participants were screened by Hamilton Depression Rating Scale (HAMD-17) and Hamilton Anxiety Rating Scale (HAMA-14) for psychological status, and Pittsburgh sleep quality index (PSQI) assessment. 409 participants were finally enrolled, of which 188 (45.97%) were suffered from sleep disorders. All participants were received polysomnography (PSG) followed by six-week pharmacological treatment of escitalopram and zopiclone, and finally assessed by HAMD-17 and HAMA-14 for treatment efficacy.

**Result:**

PSG monitoring indicated that participants with depression experienced prolonged rapid eye movement sleep latency (REMSL) and increased wakefulness after sleep onset (WASO) (P=0.030 and P=0.002, respectively). Those with anxiety disorders demonstrated a significantly higher percentage of non-rapid eye movement sleep (NREM%) and reduced WASO (P=0.013 and P=0.001, respectively). After six-weeks pharmacological treatment, participants with or without sleep disorders exhibited with similar efficacy outcomes of depression and anxiety disorders (P>0.05). However, every point of PSQI increment at baseline would decrease 0.78 and 0.85 times of probability of effective pharmacological treatment of depression and anxiety disorders. Moreover, participants with both effective outcomes of depression and anxiety disorders were found significant shorter sleep onset latency (SOL) (P<0.001).

**Discussion:**

The insights gained underscore the necessity of considering sleep disturbances in enhancing the overall effectiveness of pharmacological treatments for depression and anxiety disorders.

## 1 Introduction

Major depressive disorder (MDD) constitutes a widespread mood disorder, comprising a spectrum of conditions characterized by depressed mood and accompanied by cognitive and behavioral changes that significantly impair social functioning. According to World Health Organization (WHO) data, an estimated 280 million people worldwide are afflicted with depression, representing approximately 3.8% of the global population. Globally, adults with depression comprise about 5% of the total adult population. Patients afflicted with depressive disorders commonly experience various degrees of sleep disturbances, which consequently can aggravate the symptoms of the disorder. Between 50% and 90% of individuals with depressive disorders exhibit sleep disturbances, primarily characterized by difficulties in initiating or maintaining sleep. Research indicates that chronic insomnia heightens the risk of relapse in patients with newly diagnosed depressive disorders (1), sleep deprivation elevates the risk of postpartum depression, and poor sleep quality correlates with increased severity of depressive disorders (2).

The etiology of depressive disorders remains elusive. Current understanding suggests that depressive disorders are associated with neurotransmitter function and homeostatic imbalances, with prevailing hypotheses including the serotonin, dopamine, and norepinephrine system hypotheses. Recent research has demonstrated that biorhythm disorders, which constitute significant clinical features and pathophysiological mechanisms, are widespread among patients with depressive disorders (3–7). Biorhythm disorders are intimately linked to the onset, symptomatology, prognosis, and recurrence of depressive disorders (8–10).

Despite the frequent clinical manifestations of biorhythm disorders in patients with depressive disorders, the clinical evaluation of biorhythms is not routinely conducted (11). Although recent advancements in brain imaging, such as functional magnetic resonance imaging (fMRI), have offered new insights into the diagnosis and treatment of depressive disorders, establishing a precise correlation between imaging changes and depressive symptoms remains challenging. Furthermore, the sensitivity and specificity of these findings often fail to meet the rigorous demands of clinical diagnosis. Consequently, there is an imperative need to develop objective tools for the assessment of depressive disorders. The aim of this study is to develop a sleep rhythm evaluation system and to investigate the impact of sleep disturbances on the treatment efficacy and prognosis of depressive disorders.

Research from the Sequenced Treatment Alternatives to Relieve Depression (STAR-D) initiative has demonstrated that depression patients with characteristics such as nocturnal biorhythms, sleep rhythm disturbances, daytime hypersomnia, and daytime or seasonal affective changes typically exhibit more severe conditions, poorer treatment outcomes, increased suicide risks, greater residual symptoms, and reduced recovery rates (12–14). An investigation into the correlation between the severity of biorhythm disturbances and depressive disorders found that increased severity of rhythm disturbances is directly correlated with heightened depression severity (15).

This research primarily seeks to elucidate the relationship between sleep disturbances and symptom severity in patients with depressive and anxiety disorders, as well as the efficacy of pharmacological treatment. With the use of polysomnography (PSG), this study also explore the impact of abnormal sleep patterns on pharmacological treatment depression and anxiety.

## 2 Materials and methods

### 2.1 Participant recruitment

Between January 2021 and December 2023, 417 participants enrolled in our hospital’s Department of Psychosomatic Medicine. Inclusion criteria for participants were as follows: aged between 18 and 60 years; met the ICD-10 diagnostic criteria for depressive or anxiety disorders; scored greater than 7 points on the Hamilton Depression Rating Scale (HAMD-17) or Hamilton Anxiety Rating Scale (HAMA-14); Exclusion criteria were as follows: bipolar disorder, obsessive-compulsive disorder, post-traumatic stress disorder, restless legs syndrome, sleep apnea-hypopnea syndrome, rapid eye movement sleep behavior disorder, serious organic diseases, and alcohol or drug dependence. The ethics committee of our hospital has approved this study, and informed consent was obtained from all patients or their families.

### 2.2 Interventions and procedures

**Figure 1:**
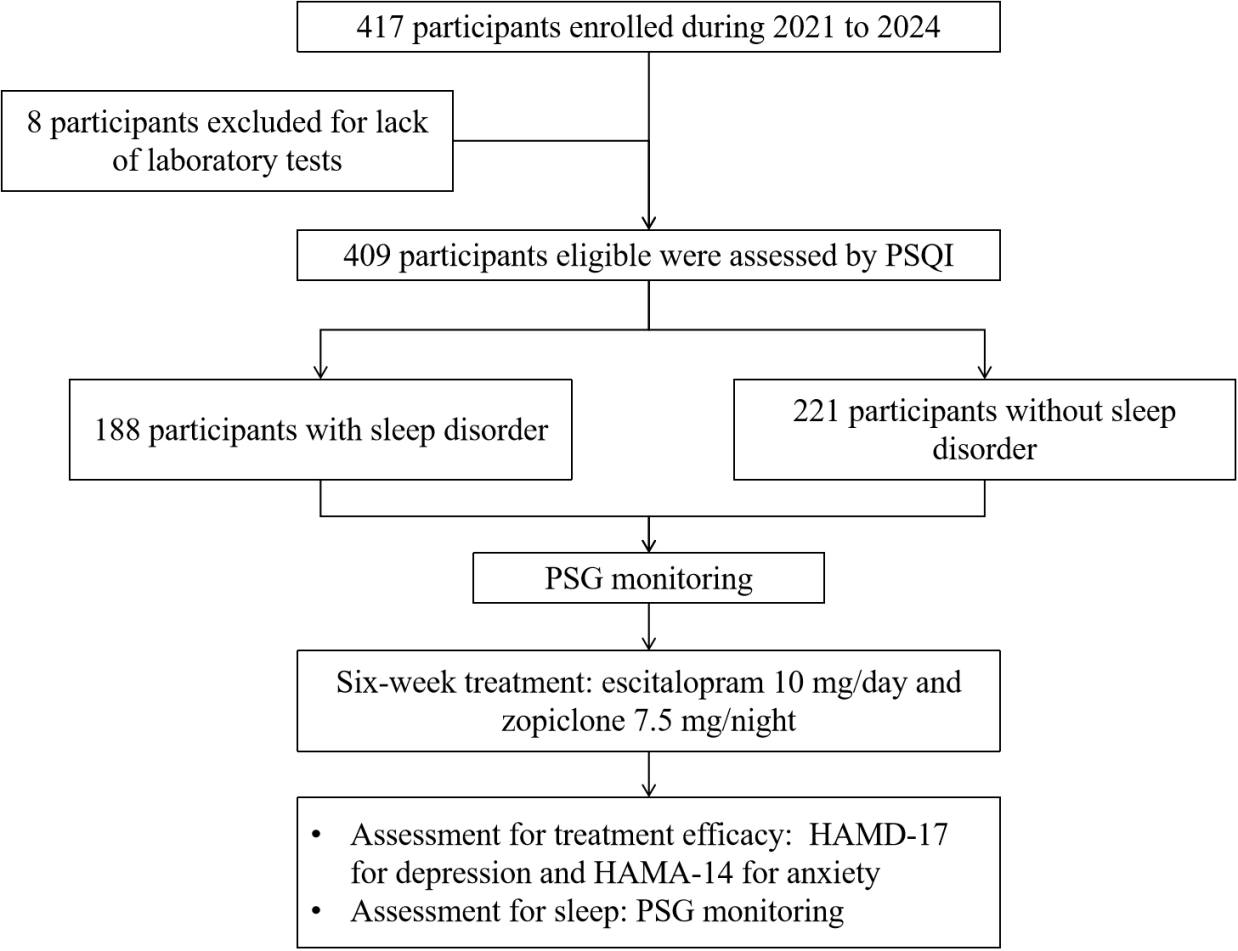
Flowchart of this study. HAMD-17, Hamilton Depression Rating Scale; HAMA-14, Hamilton Anxiety Rating Scale; PSG, polysomnography; PSQI, Pittsburgh sleep quality index.

After evaluations of age, medical histories, HAMD-17, HAMA-14, and Pittsburgh Sleep Quality Index (PSQI), eligible participants underwent overnight polysomnography. Then participants in both groups received an initial treatment of escitalopram 10 mg/day and zopiclone 7.5 mg/night, with the option to increase the escitalopram dosage to 20 mg/day after two weeks. The treatment course lasted for six weeks, during which HAMD-17 and HAMA-14 were assessed at the end of the treatment period. Study flowchart is illustrated in **Figure 1**.

### 2.3 Assessments

#### 2.3.1 Sleep rhythm assessment

Sleep disturbances were evaluated using the Pittsburgh sleep quality index (PSQI) scale, where scores ranged as follows: 0-5 denoting normal sleep quality; 6-10 suggesting a mild sleep disorder; 11-15 indicating a moderate sleep disturbance; 16-20 reflecting a severe sleep disorder; and 21 representing a very severe sleep disorder. The analysis specifically categorized sleep patterns into three types: nocturnal, intermediate, and early morning. Anxiety disorders were assessed with the HAMA-14, with scoring interpreted as follows: less than 7 indicating no anxiety, 8-13 suggesting possible anxiety, 14-20 confirming anxiety, 21-28 indicating significant anxiety, and 29 or higher denoting severe anxiety. Depressive disorders were evaluated using the HAMD-17 scale, with scores categorized as follows: 0-7 indicating no depression, 8-17 suggesting mild depression, 18-24 denoting moderate depression, and 25 or greater indicating severe depression.

#### 2.3.2 Polysomnography monitoring

Before any pharmacological treatment for depression and/or anxiety, PSG monitoring was conducted on all participants to exclude disorders such as SAHS, RLS, and rapid eye movement sleep behavior disorder. Sleep parameters including total sleep time (TST), wakefulness after sleep onset (WASO), percentage of stage 1 sleep (N1%), percentage of stage 2 sleep (N2%), percentage of stage 3 sleep (N3%), sleep onset latency (SOL), rapid eye movement sleep latency (REMSL), and percentage of REM sleep (REM%) were monitored.

#### 2.3.3 Laboratory tests

All participants were received laboratory tests from blood samples at baseline, which include triiodothyronine (T_3_), tetraiodothyronine (T_4_), free triiodothyronine (FT_3_), free tetraiodothyronine (FT_4_), thyroid stimulating hormone (TSH), thyroid peroxidase antibody (TPO_Ab_), thyroglobulin antibody (TG_Ab_), growth hormone (GH), follicle stimulating hormone (FSH), luteinizing hormone (LH), and prolactin (PRL) for endocrinological assessments, cancer antigen 125 (CA125), cancer antigen 199 (CA199), alpha-fetoprotein (AFP), carcinoembryonic antigen (CEA) as tumor biomarkers, and white blood cell (WBC) count, hypersensitive C-reaction protein (hs-CRP), smooth muscle antibody (SMA), anti-mitochondrial antibody (AMA), and anti-nuclear antibody (ANA) for immunological evaluations.

#### 2.3.4 Evaluation of efficacy

Treatment efficacy is evaluated based on the rate of score reduction of HAMA-14 or HAMD-17 scale. The criteria are as follows: a reduction rate of over 75% is considered as recovered; 50%-75% as significant improved; 25%-49% as improved; and less than 25% as ineffective. The score reduction rate is calculated using the formula: (pre-treatment score−post-treatment score)/pre-treatment score ×100%. The total effectiveness rate is calculated as: (number of recovered cases + number of significant improved cases + number of improved cases)/total number of cases × 100%.

### 2.4 Statistical analysis

For quantitative data, a Shapiro-Wilk test for normality was first conducted. Normal quantitative data were described using mean and standard deviation (SD), and examined by t-tests for two-group comparisons. Quantitative data without normality were described using median and interquartile range (P50, P25, P75). Wilcoxon rank-sum test was used for comparisons between two groups, and the Kruskal-Wallis tests were used for comparisons among multiple groups. For categorical data without order, frequencies and proportions (%) are used to represent the data, and chi-square tests are used for comparisons between two or multiple groups. For categorical data with order, rank-sum tests were used for examinations between groups. Logistic regressions were used to examined the associations between depression and anxiety treatment effectiveness and PSQI, HAMA-14, and HAMD-17 scores at baseline. P<0.05 was deemed to be significant. All analysis were performed in Stata 16.0.

## 3 Results

### 3.1 Baseline characteristics

Baseline characteristics of the study participants are summarized in **Table 1**. This study initially enrolled a total of 417 participants. Eight participants lacking blood tests were excluded, resulting in a final cohort of 409. The mean age, height, weight, and BMI of participants were 48.41 ± 0.95 years, 64.15 ± 0.50 cm, 59.56 ± 0.57 kg, and 21.97 ± 3.42 kg/m^2^, respectively. Majority of participants were female (70.90%) and married (72.37%). Among all participants, 6.11% of them were diabetic, 17.85% suffered from hypertension, 9.54% found having histories of thyroid diseases, and 9.29% diagnosed with hyperlipidemia. After HAMD-17 and HAMA-14 evaluation, 55.26% and 40.83% of participants were diagnosed with depression and anxiety disorders respectively.

**Table 1:** Baseline demographics and comorbidities in all participants and participants with or without sleep disturbances.

	All participants (n=409)	Sleep disturbances		
		No (n=221)	Yes (n=188)	*P*
**Age, years**	48.41 ± 0.95	50.73 (30.80, 63.45)	55.47 (35.33, 63.00)	0.872
**Height, cm**	164.15 ± 0.50	163.00 (158.00, 168.00)	163.00 (158.00, 170.00)	0.668
**Weight, kg**	59.56 ± 0.57	59.00 (51.00, 66.00)	57.90 (50.00, 64.96)	0.392
**BMI, kg/m^2^**	21.97 ± 3.42	22.06 (19.57, 24.19)	21.83 (19.53, 23.91)	0.271
Gender, n (%)				
Male	119 (29.10)	153 (69.23)	137 (72.87)	0.419
Female	290 (70.90)	68 (30.77)	51 (27.13)	
Marital status, n (%)				
Married	296 (72.37)	162 (73.30)	134 (71.28)	0.215
Unmarried	96 (23.47)	54 (24.43)	42 (22.34)	
Divorce	10 (2.44)	3 (1.36)	7 (3.72)	
Unknown	7 (1.71)	2 (0.90)	5 (2.66)	
Comorbidities, n (%)				
Diabetes	25 (6.11)	9 (4.27)	16 (8.51)	0.062
Hypertension	73 (17.85)	29 (13.12)	44 (23.40)	0.007
Thyroid disease	39 (9.54)	12 (5.43)	37 (19.68)	<0.001
Hyperlipidemia	38 (9.29)	9 (4.07)	27 (14.36)	<0.001
Mental disorders, n (%)				
Depression	226 (55.26)	148 (66.97)	78 (41.49)	<0.001
Anxiety	167 (40.83)	67 (30.21)	100 (53.19)	<0.001

Data are described as mean and standard deviation, and number and percentage.

BMI, body mass index.

### 3.2 Sleep disturbances analysis

No differences were observed in demographics including age, height, BMI, gender and marital status between participants with and without sleep disturbances (P>0.05). Prevalence of diabetes was found no significant difference in participants with and without sleep disturbances (8.51% vs. 4.27%, P=0.062, **Table 1**). However, hypertension (23.40% vs. 13.12%, P=0.007), thyroid disease (19.68% vs. 5.43%, P<0.001), and hyperlipidemia (14.36% vs. 4.07%, P<0.001) were more prevalent in participants with sleep disturbances than those without them (**Table 1**).

As shown in **Table 1**, significantly less participants with sleep disturbances, compared with those without sleep disturbances, were diagnosed with depression disorders (41.49% vs. 66.97%, P<0.001). While it was contrary to the distribution of diagnosis of anxiety disorders (30.21% vs. 53.19%, P<0.001).

Results of laboratory tests are summarized in **Table 2**. Significant differences were observed in the levels of tetraiodothyronine (T_4_) between participants with and without sleep disturbances (median of 110.06 nmol/L vs. 114.85 nmol/L, P=0.019). For those diagnosed with depression disorders, significant differences were noted in the levels of T_4_ (median of 114.85 nmol/L vs. 109.56 nmol/L,P=0.016), follicle stimulating hormone (FSH) (median of 16.61 U/L vs. 7.90 U/L, P=0.037), and white blood cell count (WBC) (median of 5.30 *10^9^/L vs. 5.45 *10^9^/L, P=0.029), when compared to participants both with and without these disorders. In the comparison of participants with and without anxiety disorders, significant differences were identified in the levels of T_4_ (median of 108.47 nmol/L vs. 116.78 nmol/L, P=0.005), free triiodothyronine (FT_3_) (median of 4.87 pmol/L vs. 4.97 pmol/L, P=0.036), and thyroglobulin antibody (TG_Ab_) (median of 0.00 IU/mL vs. 0.10 IU/mL, P=0.002).

**Table 2:** Analysis of laboratory tests by diagnosis of sleep disturbances, depression, and anxiety.

Parameters	Sleep disturbances			Depression			Anxiety		
	No (n=221)	Yes (n=188)	*P*	No (n=183)	Yes (n=226)	*P*	No (n=242)	Yes (n=167)	*P*
Endocrinological biomarkers									
T_3_, nmol/L	1.47 (1.29, 1.60)	1.43 (1.24, 1.58)	0.216	1.42 (1.25, 1.56)	1.47 (1.28, 1.62)	0.083	1.45 (1.27, 1.58)	1.43 (1.28, 1.60)	0.641
T_4_, nmol/L	114.85 (103.76, 127.04)	110.06 (96.05, 121.93)	0.019	109.56 (98.65, 121.24)	114.85 (102.50, 128.58)	0.016	116.78 (101.90, 128.11)	108.47 (97.70, 119.28)	0.005
FT_3_, pmol/L	4.92 (4.61, 5.36)	4.91 (4.56, 5.21)	0.117	4.89 (4.53, 5.22)	4.92 (4.66, 5.31)	0.081	4.97 (4.64, 5.35)	4.87 (4.54, 5.17)	0.036
FT_4_, pmol/L	12.24 (11.14, 13.26)	12.23 (10.76, 13.04)	0.360	12.23 (10.88, 13.03)	12.23 (11.16, 13.25)	0.737	12.19 (11.06, 13.02)	12.70 (11.02, 13.45)	0.123
TSH, μIU/mL	1.60 (1.06, 2.07)	1.38 (1.00, 1.95)	0.168	1.47 (1.05, 1.92)	1.52 (1.02, 2.14)	0.595	1.52 (1.04, 2.08)	1.44 (1.01, 1.95)	0.591
TPO_Ab_, IU/mL	0.50 (0.30, 1.20)	0.70 (0.38, 1.22)	0.441	0.60 (0.30, 1.10)	0.60 (0.32, 1.25)	0.544	0.70 (0.30, 1.30)	0.50 (0.30, 0.90)	0.261
TG_Ab_, IU/mL	0.10 (0.00, 0.20)	0.10 (0.00, 0.20)	0.795	0.10 (0.00, 0.18)	0.10 (0.00, 0.20)	0.656	0.10 (0.00, 0.20)	0.00 (0.00, 0.10)	0.002
GH, μg/L	0.37 (0.15, 0.63)	0.50 (0.18, 1.31)	0.214	0.49 (0.24, 0.93)	0.36 (0.14, 0.80)	0.076	0.40 (0.15, 1.16)	0.42 (0.16, 0.74)	0.481
FSH, U/L	12.50 (4.63, 58.66)	11.13 (4.49, 55.38)	0.637	7.90 (3.68, 40.22)	16.61 (5.21, 60.62)	0.037	11.31 (5.13, 58.69)	11.42 (3.61, 56.46)	0.526
LH, U/L	10.27 (4.38, 22.62)	10.47 (4.63, 23.66)	0.941	9.58 (3.77, 22.24)	12.44 (4.64, 23.95)	0.399	10.27 (4.67, 21.83)	10.47 (3.58, 25.87)	0.898
PRL, mU/L	368.63 (248.65, 476.61)	395.63 (273.69, 551.88)	0.159	422.95 (295.95, 574.64)	338.13 (242.66, 460.10)	0.007	393.04 (251.57, 503.13)	377.15 (267.59, 529.37)	0.774
Tumor biomarkers									
CA125	8.30 (5.63, 11.14)	7.10 (5.30, 9.38)	0.093	7.50 (4.98, 10.10)	8.00 (5.80, 10.96)	0.143	8.15 (5.63, 11.14)	7.20 (5.40, 9.66)	0.131
CA199	7.55 (4, 33, 11.20)	5.80 (3.80, 9.22)	0.082	7.65 (3.90, 10.44)	6.30 (4.00, 10.28)	0.609	7.15 (4.23, 9.30)	6.50 (3.70, 10.96)	0.589
AFP	2.40 (1.74, 2.90)	2.40 (1.74, 2.95)	0.761	2.50 (1.67, 3.21)	2.30 (1.80, 2.81)	0.480	2.32 (1.80, 2.90)	2.52 (1.70, 3.03)	0.642
CEA	1.30 (0.80, 1.80)	1.28 (0.83, 1.53)	0.315	1.30 (0.87, 5.45)	1.20 (0.77, 1.70)	0.574	1.20 (0.80, 1.62)	1, 34 (0.82, 1.69)	0.502
Immunological biomarkers									
WBC count, *10^9^/L	5.40 (4.20, 6.34)	5.32 (4.44, 6.00)	0.529	5.45 (4.57, 6.37)	5.30 (4.10, 6.00)	0.029	5.40 (4.30, 6.19)	5.33 (4.29, 6.14)	0.725
hs-CRP, mg/L	0.50 (0.50, 0.50)	0.50 (0.50, 0.53)	0.055	0.50 (0.50, 0.50)	0.50 (0.50, 0.50)	0.461	0.50 (0.50, 0.50)	0.50 (0.50, 0.51)	0.071
SMA, n (%)									
–	220 (99.55%)	187 (99.47%)	1.000	181 (98.91%)	226 (100.00%)	0.200	240 (99.17%)	167 (100.00%)	0.516
±	1 (0.45%)	1 (0.53%)		2 (1.09%)	0 (0.00%)		2 (0.83%)	0 (0.00%)	
AMA, n (%)									
–	221 (100.00%)	187 (99.47%)	0.460	183 (100.00%)	225 (99.56%)	1.000	241 (99.59%)	167 (100.00%)	1.000
+	0 (0.00%)	1 (0.53%)		0 (0.00%)	1 (0.44%)		1 (0.41%)	0 (0.00%)	
ANA, n (%)									
–	171 (55.52%)	137 (44.48%)	0.574	139 (44.74%)	226 (55.26%)	0.696	178 (57.79%)	130 (42.21%)	0.146
±	36 (49.32%)	37 (50.68%)		30 (41.10%)	43 (58.90%)		50 (68.49%)	23 (31.51%)	
+	14 (50.00%)	14 (50.00%)		14 (50.00%)	14 (50.00%)		14 (50.00%)	14 (50.00%)	

Data are described as median and interquartile, and number and percentage.

AFP, alpha-fetoprotein; AMA, anti-mitochondrial antibody; ANA, anti-nuclear antibody; CA125, cancer antigen 125; CA199, cancer antigen 199; CEA, carcinoembryonic antigen; FSH, follicle stimulating hormone; FT_3_, free triiodothyronine; FT_4_, free tetraiodothyronine; GH, growth hormone; hs-CRP, hypersensitive C-reaction protein; LH, luteinizing hormone; PRL, prolactin; SMA, smooth muscle antibody; T_3_, triiodothyronine; T_4_, tetraiodothyronine; TSH, thyroid stimulating hormone; TPO_Ab_, thyroid peroxidase antibody; TG_Ab_, thyroglobulin antibody; WBC, white blood cell.

The PSQI examination results are summarized in **Table 3**. No significant differences of laboratory tests results across PSQI scores groups were observed (P>0.05).

**Table 3:** Analysis of laboratory tests by different Pittsburgh sleep quality index scores groups.

Parameters	PSQI scores groups*				*P*
	Severe and very severe (n=141)	Moderate (n=135)	Mild (n=114)	Normal (n=19)	
Endocrinological biomarkers					
T_3_	1.40 (1.24, 1.53)	1.47 (1.31, 1.61)	1.50 (1.28, 1.63)	1.42 (1.28, 1.48)	0.156
T_4_	113.47 (101.71, 125.69)	108.75 (99.07, 124.19)	113.32 (99.51, 125.33)	108.89 (100.85, 111.90)	0.289
FT_3_	4.87 (4.63, 5.22)	4.95 (4.56, 5.27)	5.01 (4.63, 5.33)	4.64 (4.39, 4.95)	0.185
FT_4_	12.25 (11.21, 13.22)	12.25 (10.84, 13.04)	12.23 (10.92, 13.15)	12.14 (10.39, 13.73)	0.866
TSH	1.33 (0.96, 1.92)	1.55 (0.97, 2.10)	1.61 (1.08, 2.06)	1.43 (1.31, 2.19)	0.481
TPO_Ab_	0.69 (0.30, 1.35)	0.50 (0.40, 1.19)	0.60 (0.30, 1.20)	0.64 (0.40, 1.00)	0.999
TG_Ab_	0.10 (0.00, 0.10)	0.10 (0.00, 0.10)	0.10 (0.00, 0.20)	0.10 (0.00, 0.20)	0.055
GH	0.36 (0.16, 0.82)	0.51 (0.23, 1.37)	0.38 (0.14, 0.89)	0.26 (0.22, 0.55)	0.345
FSH	13.99 (4.56, 52.90)	11.22 (4.47, 59.05)	9.81 (5.01, 52.19)	13.86 (4.10, 57.71)	1.000
LH	9.88 (3.55, 21.75)	12.76 (4.59, 23.92)	7.23 (4.37, 22.48)	17.40 (8.05, 31.52)	0.515
PRL	347.26 (249.34, 499.08)	418.43 (273.61, 534.79)	377.87 (248.83, 519.82)	351.78 (273.89, 483.44)	0.691
Tumor biomarkers					
CA125	7.50 (5.00, 10.48)	8.50 (6.00, 11.30)	7.25 (5.58, 9.65)	6.50 (4.90, 9.32)	0.197
CA199	6.30 (3.90, 12.16)	6.60 (4.00, 8.70)	7.55 (3.90, 12.17)	6.70 (4.20, 9.32)	0.768
AFP	2.10 (1.60, 2.71)	2.61 (1.86, 3.08)	2.20 (1.72, 2.74)	3.11 (2.30, 3.47)	0.059
CEA	1.40 (0.81, 1.80)	1.10 (0.79, 1.50)	1.30 (0.85, 1.64)	1.45 (0.70, 1.78)	0.626
Immunological biomarkers					
WBC count, *10^9^/L	5.40 (4.30, 6.30)	5.21 (4.19, 6.00)	5.32 (4.48, 6.08)	6.30 (4.65, 7.20)	0.187
hs-CRP, mg/L	0.50 (0.50, 0.50)	0.50 (0.50, 0.50)	0.50 (0.50, 0.50)	0.50 (0.50, 0.58)	0.793
SMA, n (%)					
–	140 (99.29%)	134 (99.26%)	114 (100.00%)	19 (100.00%)	1.000
±	1 (0.71%)	1 (0.74%)	0 (0.00%)	0 (0.00%)
AMA, n (%)					
–	141 (100.00%)	134 (33.01%)	114 (100.00%)	19 (100.00%)	0.655
+	0 (0.00%)	1 (0.74%)	0 (0.00%)	0 (0.00%)
ANA, n (%)					
–	100 (70.92%)	103 (76.30%)	90 (78.95%)	15 (78.95%)	0.515
±	33 (23.40%)	22 (16.30%)	15 (13.16%)	3 (15.79%)
+	8 (5.67%)	10 (7.40%)	9 (7.89%)	1 (5.26%)

Data are described as median and interquartile, and number and percentage.

AFP, alpha-fetoprotein; AMA, anti-mitochondrial antibody; ANA, anti-nuclear antibody; CA125, cancer antigen 125; CA199, cancer antigen 199; CEA, carcinoembryonic antigen; FSH, follicle stimulating hormone; FT_3_, free triiodothyronine; FT_4_, free tetraiodothyronine; GH, growth hormone; hs-CRP, hypersensitive C-reaction protein; LH, luteinizing hormone; PRL, prolactin; PSQI, Pittsburgh sleep quality index; SMA, smooth muscle antibody; T_3_, triiodothyronine; T_4_, tetraiodothyronine; TSH, thyroid stimulating hormone; TPO_Ab_, thyroid peroxidase antibody; TG_Ab_, thyroglobulin antibody; WBC white blood cell.

*PSQI scores: 0-5 denoting normal sleep quality; 6-10 suggesting a mild sleep disorder; 11-15 indicating a moderate sleep disturbance; 16-20 reflecting a severe sleep disorder; and 21 representing a very severe sleep disorder.

### 3.3 Polysomnography results

Results of PSG monitoring results from participants diagnosed with depression and anxiety are detailed in **Table 4**. PSG monitoring demonstrated significant longer in REMSL (median of 223 min vs. 193 min, P=0.030) and WASO (median of 25 times vs. 18 times, P=0.002) in participants with depression disorders than their counterparts. For those with anxiety disorders, significant higher NREM% were found, compared to those without anxiety disorders (median of 10.20% vs. 6.20%, P=0.013). Additionally, the WASO (median of 17 times vs. 25 times, P=0.001) was significantly less, and N2% (median of 64.00% vs. 66.75%, P=0.023) was significantly lower in participants with anxiety compared to those without anxiety disorders.

**Table 4:** Comparison of polysomnography parameters of clinical diagnosis of depression and anxiety.

PSG parameters	Depression			Anxiety		
	No	yes	P	No	Yes	P
TST, min	430.00 (344.50, 464.30)	424.75 (342.88, 465.20)	0.965	430.25 (353.88, 463.80)	420.00 (338.00, 465.10)	0.420
SOL, min	17.50 (8.00, 34.50)	21.00 (9.00, 35.00)	0.523	18.25 (8.38, 32.05)	20.50 (9.50, 41.80)	0.072
REMSL, min	193.00 (129.75, 263.32)	223.00 (144.75, 300.75)	0.030	214.00 (145.50, 283.00)	193.50 (133.50, 280.00)	0.486
REM%, %	10.95 (5.45, 14.70)	8.70 (3.53, 13.00)	0.087	9.30 (3.90, 13.30)	10.70 (4.85, 14.50)	0.252
NREM%, %	8.90 (0.65, 15.04)	7.50 (0.43, 15.70)	0.995	6.20 (0.00, 14.80)	10.20 (1.88, 16.49)	0.013
WASO, times	18 (11, 29)	25 (14, 38)	0.002	25 (14, 36)	17 (10, 28)	0.001
N1%, %	9.40 (5.90, 15.78)	10.55 (6.35, 17.92)	0.104	10.05 (6.35, 15.47)	10.00 (6.00, 18.00)	0.576
N2%, %	66.10 (56.20, 72.56)	65.50 (53.50, 73.29)	0.598	66.75 (57.00, 74.02)	64.00 (50.20, 70.76)	0.023
N3%, %	8.70 (0.50, 14.00)	7.25 (0.38, 15.00)	0.828	6.20 (0.00, 14.00)	9.00 (1.50, 15.06)	0.055

Data are described as median and interquartile.

N1%, percentage of stage 1 sleep; N2%, percentage of stage 2 sleep; N3%, percentage of stage 3 sleep; PSG, polysomnography; REMSL, rapid eye movement sleep latency; REM%, percentage of REM sleep; SOL, sleep onset latency; TST, total sleep time; WASO, wakefulness after sleep onset.

**Figure 2:**
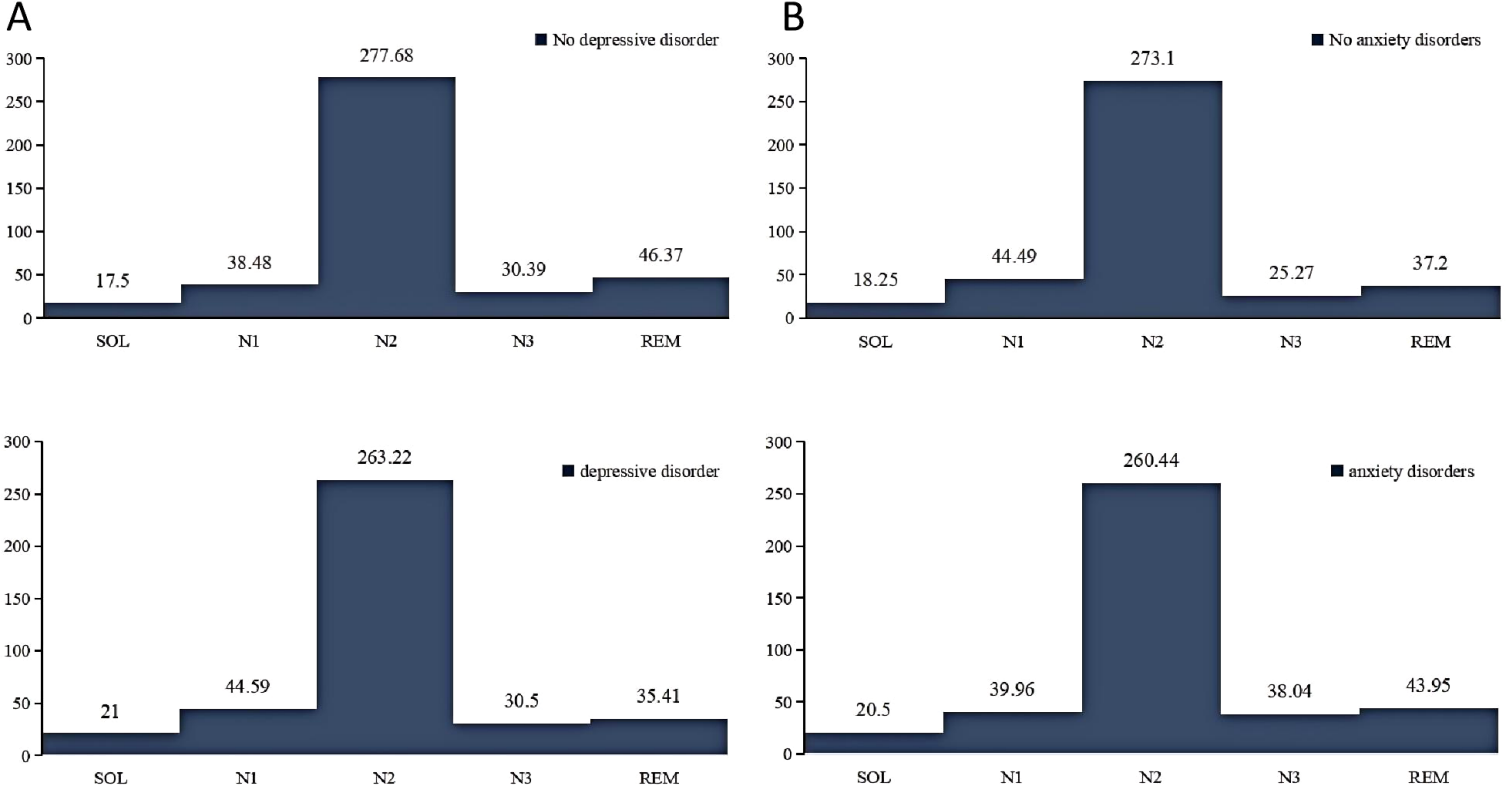
Comparison of sleep cycles in different mental disorders. **(A)**, sleep cycles in participants with and without depression. **(B)**, sleep cycles in participants with and without anxiety. N1, stage 1 sleep; N2, stage 2 sleep; N3, stage 3 sleep; REM, rapid eye movement; SOL, sleep onset latency.

**Figure 2** illustrates the comparative analysis of sleep cycles among different populations. It was observed that individuals with anxiety disorders exhibited longer SOL, and extended REM and N3 sleep stages, while their N1 and N2 stages were shorter compared to those without anxiety disorders. In contrast, individuals with depressive disorders demonstrated prolonged SOL and increased durations of N1 and N3 sleep stages, but reduced REM duration compared to those without depressive disorders.

### 3.4 Relationship between sleep disturbances and treatment outcomes

There was no significant difference after six-week pharmacological treatment outcomes of neither depression (P=0.904) nor anxiety (P=0.509) between the two groups (**Table 5**). In the group without sleep disturbances, 36 participants were recovered from depression by assessment of HAMD-17 scale, 77 were significant improved, 49 improved, and 59 showed ineffective. Conversely, in the group with sleep disturbances, 21 participants were recovered, 67 significantly improved, 44 improved, and 56 showed ineffective. Outcomes of anxiety for participants with or without sleep disturbances were as follows: recovered (34 participants vs. 33 participants), significantly improved (68 vs. 65), improved (47 vs. 75), ineffective (39 vs. 48).

**Table 5:** Comparison of clinical efficacy of six-week treatment between participants with and without sleep disturbances.

Disorders	Treatment outcomes	Sleep disturbances		P
		No (n=221)	Yes (n=188)	
**Depression**	Recovered, n	36	21	0.904
	Significantly improved, n	77	67	
	Improved, n	49	44	
	Ineffective, n	59	56	
	Effective rate	73.30%	70.21%	
**Anxiety**	Recovered, n	33	34	0.509
	Significantly improved, n	65	68	
	Improved, n	75	47	
	Ineffective, n	48	39	
	Effective rate	78.28%	79.73%	

**Table 6** presents the baseline PSG results by treatment outcomes of depression and anxiety. Patients with depression were categorized into effective and ineffective treatment groups based on their therapeutic responses. Participants with effective outcomes of both depression (median of 17.50 min vs. 30.00 min, P<0.001) and anxiety (median of 17.00 min vs. 34.50 min, P<0.001) demonstrated a significantly shorter SOL than the ineffective group. REMSL was significantly longer in participants with effective anxiety treatment outcomes compared to the ineffective group (median of 216.75 min vs. 187.25 min, P=0.034).

**Table 6:** Comparison of polysomnography parameters by six-week treatment outcomes of depression and anxiety.

PSG parameters	Depression			Anxiety		
	Ineffective	Effective	P	Ineffective	Effective	P
TST, min	430.00 (344.00, 482.00)	426.75 (343.00, 474.75)	0.534	400.00 (342.50, 477.00)	432.00 (344.88, 474.25)	0.136
SOL, min	34.50 (13.50, 53.00)	17.00 (8.00, 34.50)	<0.001	30.00 (13.50, 59.00)	17.50 (7.50, 36.50)	<0.001
REMSL, min	184.00 (136.00, 299.00)	217.50 (142.00, 304.00)	0.249	187.25 (81.88, 266.00)	216.75 (147.75, 307.13)	0.034
REM%, %	10.60 (5.00, 16.50)	9.90 (4.35, 14.78)	0.353	9.70 (3.88, 16.50)	10.00 (4.60, 14.90)	0.619
NREM%, %	10.10 (0.33, 19.63)	8.00 (0.50, 16.45)	0.339	8.10 (0.10, 19.00)	8.65 (0.58, 16.90)	0.655
WASO, times	21.00 (12.00, 38.00)	22.00 (12.00, 38.00)	0.970	24.00 (12.00, 42.00)	21.00 (12.00, 37.25)	0.712
N1%, %	9.70 (5.40, 18.35)	10.10 (6.13, 19.83)	0.431	10.40 (5.50, 19.40)	9.85 (6.28, 19.23)	0.736
N2%, %	66.70 (54.10, 73.10)	65.60 (55.00, 76.00)	0.996	65.50 (50.10, 72.70)	66.05 (55.30, 75.23)	0.414
N3%, %	7.70 (0.00, 18.45)	8.05 (0.53, 15.58)	0.977	7.30 (0.00, 18.20)	8.05 (0.58, 15.60)	0.949

Data are described as median and interquartile.

N1%, percentage of stage 1 sleep; N2%, percentage of stage 2 sleep; N3%, percentage of stage 3 sleep; PSG, polysomnography; REMSL, rapid eye movement sleep latency; REM%, percentage of REM sleep; SOL, sleep onset latency; TST, total sleep time; WASO, wakefulness after sleep onset.

Associations between effective treatment and severity of symptoms of sleep disturbances, depression, and anxiety at baseline were examined by Logistic regressions analysis in **Table 7**. PSQI scores at baseline were negatively associated with effective treatment of both depression and anxiety with the odds ratio (OR) of 0.78 (95% of confidence interval [CI]: 0.71-0.84, P<0.001) and 0.85 (95% CI: 0.79-0.91, P<0.001), respectively, which meant every point of PSQI increment at baseline would decrease 0.78 and 0.85 times of probability of effective treatment of depression and anxiety disorders. HAMD-17 (OR: 1.19, 95% CI:1.12-1.27, P<0.001) and HAMA-14 (OR: 1.17, 95% CI:1.11-1.25, P<0.001) scores were respectively associated with effective treatment of depression and anxiety. However, no significant correlations were found neither between HAMD-17 and effective treatment of anxiety, nor HAMA-14 and depression.

**Table 7:** Associations between effective treatment and severity of symptoms of sleep disturbances, depression, and anxiety at baseline.

Baseline assessments	Effective treatment of depression		Effective treatment of anxiety	
	OR (95% CI)	P	OR (95% CI)	P
PSQI scores^*^	0.78 (0.71-0.84)	<0.001	0.85 (0.79-0.91)	<0.001
**HAMD-17 scores^†^**	1.19 (1.12-1.27)	<0.001	0.97 (0.92-1.01)	0.166
**HAMA-14 scores^‡^**	0.99 (0.94-1.04)	0.747	1.17 (1.11-1.25)	<0.001

CI, confidence interval; HAMD-17, Hamilton Depression Rating Scale; HAMA-14, Hamilton Anxiety Rating Scale; OR, odds ratio; PSQI, Pittsburgh sleep quality index.

^*^Adjusted for age, gender, BMI, comorbidities, HAMD-17, and HAMA-14.

**^†^** Adjusted for age, gender, BMI, comorbidities, HAMA-14, and PSQI scores.

**^‡^** Adjusted for age, gender, BMI, comorbidities, HAMD-17, and PSQI scores.

## 4 Discussion

The current study include 409 participants diagnosed with depression and/or anxiety disorders. comorbidities, endocrinological, tumor and immunological biomarkers were compared in participants with or without sleep disturbances, depression, and anxiety disorders. Moreover, relationships between sleep disturbances and depression as well as anxiety disorders were studied, as well as the influence of sleep disturbances and PSG monitoring results on depression and/or anxiety disorders pharmacological treatment. We found that baseline status of sleep disturbances had no impact to pharmacological treatment outcomes of both depression and anxiety disorders, which were, however, associated with PSQI scores and SOL by PSG monitoring at baseline.

Depressive symptoms are associated with a significant disease burden in adults and can contribute to premature mortality (16). This study indicates a robust association between sleep disturbances and various psychosomatic disorders, including behavioral disorders, substance disorders, and particularly anxiety disorders (17–20). Prior research has demonstrated that both PSG and multi-sensor trackers are effective in monitoring sleep patterns, although PSG is found inadequate for classifying the severity of sleep apnea in infants (21–23). Consequently, this study used PSG to monitor sleep patterns in adults diagnosed with depression and anxiety disorders. It was found that adults with depressive disorders exhibited greater difficulties in initiating sleep and had extended durations of REM sleep latency (REMSL) and frequent wake after sleep onset (WASO) compared to healthy controls (24–26). Jorge Mendoza’s research demonstrated that circadian rhythm disruptions in mood disorder patients could serve as crucial biomarkers for the prevention and treatment of depression (27).

This study sought to investigate the potential contributory role of sleep disturbances and depression in cancer risk by analyzing levels of CA125, CA199, AFP, and CEA. However, the results indicated no association between depression and these biomarker levels in cases of sleep disturbances like previous studies (28, 29). Other studies have revealed that inadequate or prolonged sleep, insomnia symptoms, and nocturnal phenotypes elevate the risk of lung cancer. Additionally, variations in sleep duration, the use of sleep aids, and insomnia manifest differently in women’s breast tissues, potentially exacerbating difficulties in sleep initiation among cancer patients (28, 29). Research has identified sleep disturbances as a cause for alterations in inflammatory factors, thereby elevating hs-CRP levels, a finding that is consistent with other studies (30, 31). Treatment options for sleep rhythm disorders encompass light therapy and techniques to counteract early rhythm phase delays (32–34). We recommend increasing physical activity to enhance mental health, as supported by previous findings (35, 36).

## 5 Limitation

Some participants in the study had incomplete test results, which were treated as missing data. Despite prior training, maintaining consistency in the investigators’ expertise level throughout the survey implementation proved challenging. This study was conducted over three years, and the investigators’ experience may deepened, potentially influencing assessment of scales and resulting in bias of the outcomes.

## 6 Conclusion

Findings from PSG revealed that participants with depression and anxiety disorders exhibited distinct sleep patterns, such as prolonged REMSL and altered WASO, which differed significantly from those without these disorders. Notably, effective pharmacological treatment outcomes were associated with significantly shorter SOL. These results revealed the importance of integrating sleep management into therapeutic strategies for mental disorders. The insights gained underscore the necessity of considering sleep disturbances in enhancing the overall effectiveness of pharmacological treatments for depression and anxiety disorders.

## Data Availability

The original contributions presented in the study are included in the article/supplementary material. Further inquiries can be directed to the corresponding authors.
